# Caffeine Administration in Piglets with Low Birthweight and Low Vitality Scores, and Its Effect on Physiological Blood Profile, Acid–Base Balance, Gas Exchange, and Infrared Thermal Response

**DOI:** 10.3390/ani13223491

**Published:** 2023-11-12

**Authors:** Dina Villanueva-García, Marcelo Ghezzi, Patricia Mora-Medina, Ismael Hernández-Ávalos, Adriana Olmos-Hernández, Alejandro Casas-Alvarado, Karina Lezama-García, Adriana Domínguez-Oliva, Daniela Rodríguez-González, Miriam Marcet-Rius

**Affiliations:** 1 Division of Neonatology, National Institute of Health, Hospital Infantil de México Federico Gómez, Mexico City 06720, Mexico; dinavg21@yahoo.com; 2 Animal Welfare Area, Faculty of Veterinary Sciences (FCV), Universidad Nacional del Centro de la Provincia de Buenos Aires (UNCPBA), University Campus, Tandil 7000, Argentina; 3 Facultad de Estudios Superiores Cuautitlán, Universidad Nacional Autónoma de México (UNAM), Cuautitlán Izcalli 54714, Mexico; 4 Division of Biotechnology-Bioterio and Experimental Surgery, Instituto Nacional de Rehabilitación Luis Guillermo Ibarra Ibarra (INR-LGII), Mexico City 14389, Mexico; 5 Neurophysiology, Behavior and Animal Welfare Assesment, DPAA, Xochimilco Campus, Universidad Autónoma Metropolitana, México City 04960, Mexicokikislezama@hotmail.com (K.L.-G.);; 6 Department of Animal Behaviour and Welfare, Research Institute in Semiochemistry and Applied Ethology (IRSEA), 84400 Apt, France

**Keywords:** caffeine, newborn piglet, blood profile, vitality score, acid–base imbalance

## Abstract

**Simple Summary:**

Intrapartum mortality reaches high figures in piglets, and it has been seen that those born with low weight and those with low or failed vitality scores are the most prone. Caffeine is used to reduce cardiorespiratory problems in neonates, both humans and animals. For this reason, the decision was made to use different doses of caffeine in newborn piglets in this study, to define if its oral administration would help reduce this problem and what would be the optimal dose and time of administration. The following blood gas parameters were evaluated: pH, pO_2_, pCO_2_, HCO_3_, Ca++, glucose, and lactate, as well as superficial and rectal temperature at 1 h, 6 h, and 24 h after birth. It was concluded that the optimal dose of caffeine to improve the vitality score and reduce possible cardiorespiratory problems is 30 mg/kg orally.

**Abstract:**

Intrapartum asphyxia, fetal hypoxia, and their consequences (e.g., acidosis, hypercapnia, hypoglycemia, and hypothermia) are the main factors related to physio-metabolic imbalances that increase neonatal mortality in piglets, particularly in piglets with low birthweight and low vitality scores. This study aimed to evaluate the effect of three different doses of caffeine (10, 20, and 30 mg/kg) administered orally to 480 newborn piglets with low birthweight and low vitality scores. Blood gas parameters (pH, pO_2_, pCO_2_, and HCO_3_^−^), physio-metabolic profile (Ca^++^, glucose, and lactate), and the thermal response assessed through infrared thermography in four thermal windows (ocular, auricular, snout, and hindlimb) and rectal temperature were evaluated during the first 24 h of life. Doses of 30 mg/kg resulted in significant differences at 24 h for all evaluated parameters, suggesting that caffeine administration improved the cardiorespiratory function and metabolic activity of piglets by reducing acidosis, restoring glycemia, and increasing surface and rectal temperature. In conclusion, caffeine at 30 mg/kg could be suggested as an appropriate dose to use in piglets with low birthweight and low vitality scores. Future research might need to study the presentation of adverse effects due to higher caffeine concentrations.

## 1. Introduction

Newborn mortality in piglets ranges from 15 to 70% during the first three days post-farrowing [1], and from 11 to 20% in the pre-weaning period [2]. Several factors influence the high mortality rates. For example, physiological immaturity at birth, intrauterine growth restriction, and dystocic farrowing [3]. The incidence of dystocia (ranging from 12.78 to 75.3%) [4,5] can result in decreased maternal blood supply to the fetus due to inadequate uterine contractions, umbilical cord rupture, or meconium consumption, leading to intrapartum asphyxia and fetal hypoxia [6,7].

As a result of fetal hypoxia, newborn piglets can be born with low vitality scores due to a decreased cardiorespiratory function that will consequently cause physio-metabolic alterations such as respiratory/metabolic acidosis (increase in lactic acid production) and hypercapnia (low O_2_ and high CO_2_ concentrations) [8,9]. Low vitality during the first 72 h post-farrowing is also associated with 2 to 30% of neonatal deaths because piglets have delayed access to the udder and are not able to ingest colostrum [10,11]. Moreover, the number of piglets is higher than the number of teats of the sow due to genetic selection; therefore, the weakest piglets have few possibilities to consume colostrum. Since piglets are born with limited glycogen reserves that are rapidly depleted in the first 12 h (by 42%) [3,12,13], inadequate colostrum intake within the first day of life predisposes piglets to starvation, hypoglycemia, and hypothermia [3,14,15].

Newborn piglets are susceptible to hypothermia due to thermoregulatory impairments related to low birth weight—limiting the energy reserves of piglets [16,17]—or to anatomical traits of the species (e.g., low hair density, limited deposits of brown adipose tissue and glycogen) [18,19,20]. Thermogenesis through shivering and brown adipose tissue, or sympathetic responses such as vasoconstriction to reduce heat loss, are mechanisms triggered by piglets to maintain their body temperature [18]. 

Caffeine administration has been suggested as a method to improve piglet neonatal survival [10]. Caffeine is a stimulant of the central nervous system (CNS) due to its adenosine receptor antagonism [21,22,23]. In humans, caffeine increases respiratory volume, decreasing hypoxia and possibly increasing newborn vitality [24,25,26]. In contrast, in veterinary medicine, the relation between the respiratory stimulatory effect and the improvement in vitality when using caffeine has not been completely established, since there is no defined therapeutic dose to improve the vigor of newborn animals with low birthweight [10,27]. Nonetheless, different doses (e.g., 10–27 mg/kg) of caffeine have recently been studied to reduce neonatal cardiorespiratory alterations [28,29,30,31]. In a study carried out in piglets by Jarrat el al. [10], they administered three treatments (caffeine 30 mg, glucose 300 mg and caffeine with glucose combined) and observed that said administration did not cause changes in the performance of the piglets evaluated with the exception of low birthweight piglets, whose growth rates improved during the first 3 days of life. 

Although different studies have assessed the effect of different doses of caffeine on parameters such as triglycerides, cholesterol, lactate, glucose, and rectal temperature [10,32], or have used different routes of administration (e.g., subcutaneous) [33], there are no studies to date associating the physio-metabolic profile, acid–base balance, and blood gas exchange parameters with the thermal response of piglets using infrared imaging. For this reason, the objective of this study was to evaluate the effect of three different doses of caffeine administered orally in newborn piglets with low birthweight and low vitality scores. The effect of caffeine on the metabolic profile, acid–base balance, blood gas exchange, and thermal response was studied using infrared imaging. We hypothesized that the administration of caffeine at a dose of 30 mg/kg would improve the biomarkers associated with the energy metabolism, gas exchange, and thermal response of newborn piglets.

## 2. Materials and Methods 

### 2.1. Study Location

The present study was performed on a commercial swine farm located in the central region of Mexico. The swine farm employs a production regimen with approximately 300 farrows per week in an ‘all in–all out’ system.

### 2.2. Animals and Housing Conditions 

4456 piglets born from 400 multiparous Landrace X Yorkshire sows were monitored before selecting the piglets for the present study, of which 480 were selected. The sow’s parity ranged from two to seven. The sows were individually housed in non-plasticized flooring crates five days before the expected farrowing date, where they stayed for 28 days until weaning. The farrowing facilities included electronic ventilation systems, with both natural and artificial lighting available. The average room temperature of the sow house was maintained at 25 °C with a relative humidity of 62%. Lighting intensity was set at 38.7 candela. The design and specifications of the farrowing facilities were in accordance with the Mexican Official Standard NOM-062-ZOO-1999 [34]. After farrowing, the sows were fed 3.2 kg of a commercial concentrated lactating animal feed (12.2 MJ ME/kg and 15% CP).

Farrowing was induced with intramuscular administration of prostaglandin (Lutalyse; Pharmacia & Upjohn, Mexico), injected 36 h before the expected farrowing date (at 114 days of gestation). On the day of farrowing, sows were not provided feed but had unrestricted access to water. No obstetrical manipulation was performed during farrowing, but there was medical intervention and resuscitation of asphyxiated piglets. Sows that experienced dystocia and required oxytocin administration or medical intervention were excluded from the study. 

### 2.3. Inclusion Criteria

Piglets with a low vitality score (≤5 points) and low birthweight (<1 kg) were included in the present study. To select the piglets, within the first minute after birth, vitality was assessed using Zaleski and Hacker’s [35] scale, modified by [36]. Five variables were assessed: heart rate, latency to respiration, latency to stand up, skin color of the snout, and meconium staining. Using a 0–10 scale, piglets with 8–10 points were classified as high vitality, 6–7 as medium vitality, and below 5 as low vitality [9].

To obtain the birthweight, piglets were weighted with a digital scale (Bascule Salter Weight-Tronix, West Bromwich, UK) after vitality assessment. A total of 480 piglets were selected for the evaluation of physiological and thermal parameters. 

### 2.4. Treatments

The 480 newborn piglets were randomly assigned to four treatments (*n* = 120 piglets in each group). To establish the different doses, 0.1 mL (low dose), 0.2 mL (medium dose), and 0.3 mL (high dose) of caffeine (Loeffler Laboratories, Mexico, 100 mL/10 g of caffeine. Each milliliter contains 100 mg of caffeine) were diluted in 6 mL of 9% saline solution (Pisa Laboratories, Mexico City, Mexico). The treatments were divided as follows: placebo (G_1_): oral administration (PO) of 6 mL of saline per piglet; low dose of caffeine (G_2_), 10 mg/kg PO; medium dose of caffeine (G_3_), 20 mg/kg PO; and high dose of caffeine (G_4_), 30 mg/kg PO. PO administration was performed using a syringe, briefly restraining the piglet to ensure that the entire dose of the drug was taken. 

### 2.5. Experimental Design

Neonatal monitoring was performed by four trained D.V.M. M.Sc. Immediately after birth and after vitality and birthweight recording, piglets were completely dried with kraft paper, rubbing the piglets for 2 min. After drying the animals, thermal imaging and blood sampling were performed in four evaluation times by two blind evaluators: basal: before the PO administration of caffeine, after piglets were dried; 1 h: one hour after birth; 6 h: six hours after birth; and 24 h: 24 h after birth.

All newborn piglets were managed according to standard farm protocols, including umbilical cord cutting and colostrum intake within the first hour after birth. Piglets were identified using an indelible marker on the dorsal skin. At basal, 1 h, 6 h, and 24 h, thermal imaging was performed before retro-orbital sinus (ROS) bleeding to avoid handling stress due to blood sampling. Likewise, during the 24 h of the experimental phase, none of the piglets was subjected to routine farm practices such as tail docking, iron injection, or ear tagging. These routine procedures were performed at three days old, after the study’s completion.

### 2.6. Assessed Variables

#### 2.6.1. Thermal Imaging

**Figure 1:**
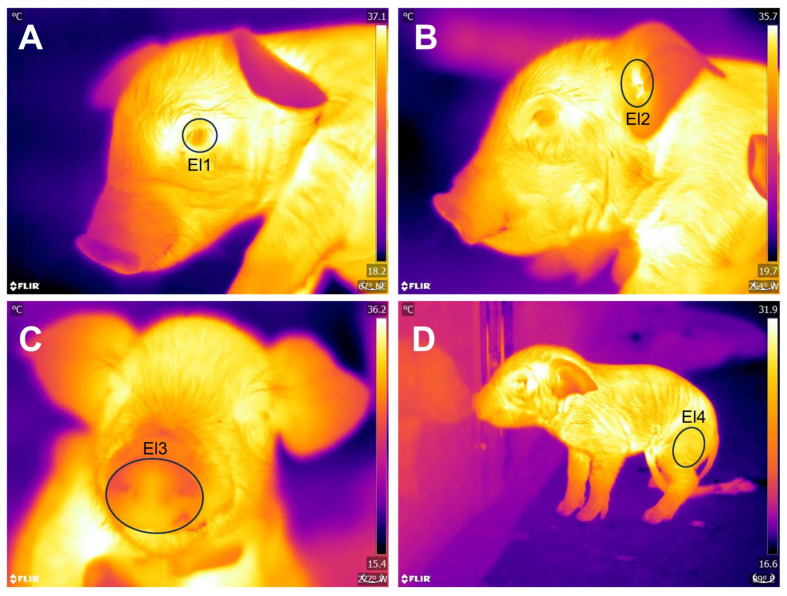
Delimitation of thermal windows. (**A**) OCULAR (El1). (**B**) EAR (El2). (**C**) NOSE (El3). (**D**) LIMB (El4).

An infrared thermal camera FLIR^®^ E60 (FLIR Systems, Wilsonville, OR, USA) was used. The camera had a thermal sensibility of <0.045 °C, an IR resolution of 320x240 pixels, and a precision of ± 2 °C or 2%. Thermal imaging was performed at a maximum distance of 1 m from the piglets, focusing on four thermal windows: the periocular area (*Regio orbitalis*) (OCU), delimited by an ellipse covering the entire ocular surface, including a few millimeters of the upper and lower eyelids; the auricular region (*Regio auricularis*) (EAR), set by an ellipse in the central cartilage; the nasal region (*Regio nasalis*) (NOSE), drawn as a circle including the snout, using the upper lip as a ventral limit; and the left hindlimb (LIMB), at the edge of the pelvic limb, in the biceps femoris region. Figure 1 shows the delimitation of said thermal windows. 

During basal, 1 h, 6 h, and 24 h, IRT was performed before handling the piglets for blood sampling. All images were saved in JPG format to analyze them in FLIR Tools (FLIR Systems, USA) software, obtaining the maximum, minimum, and average values.

#### 2.6.2. Blood Gas and Physiological Profile 

At all evaluation times (basal, 1 h, 6 h, and 24 h), 150 µL of blood was collected from the ROS with lithium heparin capillary tubes. Immediately after sampling, on the farm, all samples (150 µL) were processed in a GEM Premier blood gas analyzer (Instrumentation Laboratory Diagnostics, Lexington, KY, USA/Milan, Italy) to analyze the following metabolites: glucose (mg/dL), lactate (mg/dL), partial pressure of carbon dioxide (pCO_2_) (mmHg), partial pressure of oxygen (pO_2_) (mmHg), pH, bicarbonate (HCO_3_^-^) (mmol/L), and Ca^++^ (mmol/L). 

### 2.7. Statistical Analysis

The analysis of the data was performed using the statistical package GraphPad Prism (Ver. 9.4.0). Descriptive statistics were obtained for each variable of G_1_, G_2_, G_3_, and G_4_. A Shapiro–Wilk test was performed to analyze the normality of the data.

The data of blood gases, physio-metabolic blood profile, and surface temperature of the different thermal windows were considered independent variables, while the caffeine dose was considered a dependent variable. To assess the effects of these variables, an analysis of variance (ANOVA) under a mixed linear model was used:

Yijk= µ+τi+τj + τiτj + βk +eij

where Y = response variable gasometry, physio-metabolic blood profile, and superficial thermal response. τi = effect of caffeine dose (G_1_, G_2_, G_3_, and G_4_). τj = effect of the evaluation time (basal, 1 h, 6 h, and 24 h). β = random effect (animal). µ = population mean. e; = residue.

The correlations between the rectal temperature and the IRT temperature were analyzed using a Pearson correlation. To evaluate the differences between the means, a Tukey post hoc test was used. In all cases, a significance level of *p* < 0.05 was set. Under this same model, the effect of sex on each treatment (G_1_, G_2_, G_3_, and G_4_) was evaluated.

### 2.8. Ethical Statement

The experimental procedure of the current investigation received approval from the Laboratory of Animal Ethology and Neurophysiology (ETO-078-21) and the Postgraduate Veterinary Commission of the Universidad Autónoma Metropolitana in Mexico City, Mexico. The management of the animals was in accordance with FRAME’s guidelines for animal research and the ethical considerations for applied ethological studies [37]. Acknowledging that the procedures of the present study might cause mild pain, suffering, and/or distress, the use of cell phones, televisions, and other sources of noise was restricted to minimize stressors. All animals were treated with humane care. The design and specifications of the farrowing facilities were in accordance with the Mexican Official Standard NOM-062-ZOO-1999 [34].

## 3. Results

Differences in blood gas and physio-metabolic parameters were observed in all newborn piglets according to the caffeine dose. In particular, caffeine at a dose of 30 mg/kg (G_4_) registered significant differences and the highest changes from basal to 24 h. Regarding the effect of sex in the piglets, no significant differences were found in the present study. Therefore, results are presented as pooled data from males and females, expressed as mean ± standard error (SE).

### 3.1. Blood Gas Parameters 

Table 1 shows the values of blood gas parameters (pH, pCO_2_, pO_2_, and HCO_3−_) and the pH values of the four experimental groups. When comparing G_2_ and G_3_ with G_4_, it can be observed that the pH of G_4_ piglets was significantly higher (*p* = 0.001) at 1 h, 6 h, and 24 h by 0.03, 0.01 and 0.09, respectively. Likewise, G_4_ significantly differed from basal to 1 h, 6 h, and 24 h (*p* = 0.001), recording progressive increases of 0.01, 0.11, and 0.14, respectively.

Regarding pCO_2_, the G_4_ dose of caffeine significantly reduced pCO_2_ by 4 mmHg, 3 mmHg, and 22.57 mmHg at 1 h (*p* = 0.0001), 6 h (*p* = 0.001), and 24 h (*p* = 0.001), respectively, compared with G_2_ and G_3_. The same experimental group (G_4_) had significant reductions of up to 27.03 mmHg at 24 h (*p* = 0.001).

In contrast to pCO_2_ in G_4_, pO_2_ increased significantly from 16.44 ± 0.17 mmHg at 1 h after administration of caffeine to 21.06 ± 0.23 mmHg at 24 h (*p* = 0.0005) (Table 1). A comparison between evaluation times showed that, while G_2_ piglets increased their pO_2_ by 2.85 mmHg from basal to 1 h, they had a significant decrease of 2 mmHg from 1 h to 6 h (*p* = 0.0001). In contrast, a progressive increase was observed in G_3_ and G_4_, increasing up to 4 mmHg and 4.62 mmHg, respectively (*p* = 0.0001).

When addressing HCO_3_^-^, similarly to other blood parameters, piglets in G_4_ had the highest increases compared to G_2_ and G_3_, registering increases of 1.06 mmol/L at 6h (*p* = 0.0001) and 2.8 mmol/L at 24 h (*p* = 0.0001). Between evaluation times, all groups receiving caffeine increased their HCO_3_^-^ levels by 1.22 mmol/L in G_2_ (*p* = 0.001), 2.21 mmol/L in G_3_ (*p* = 0.001), and 4 mmol/L in G_4_ from basal to 24 h.

### 3.2. Physio-Metabolic Profile

The differences between treatments and Ca^++^ values are presented in Table 2. In G_2_, G_3_, and G_4_, caffeine significantly reduced Ca^++^ by 8%, 12%, and 14%, respectively, at 24 h (*p* = 0.002). In particular, G_4_ registered a significant decrease from basal (2.08 ± 0.01 mmol/L) to 24 h (1.80 ± 0.01 mmol/L) (*p* = 0.0001). For glucose, G_4_ showed a statistically significant increase of 1-2 mg/dL at 1 h (*p* = 0.0001) and a similar increase at 6 h *(p* = 0.0001). At 24 h, although all groups showed increasing values, G_4_ registered the highest glucose concentration of 97.33 ± 0.70 mg/dL (*p* = 0.007). Similarly, it was observed that only G_4_ registered a statistically significant progressive increase of 12.28 mg/dL at 1 h, 15.28 mg/dL at 6 h, and 40.25 mg/dL at 24 h (*p* = 0.02)

The differences found in lactate values (presented in Table 2), show that, when comparing G_2_ and G_3_, lactate concentration significantly decreased by 15 mg/dL and 8 mg/dL, respectively, at 6 h, and by 42.75 mg/dL and 33.59 mg/dL, respectively, at 24 h. For all groups, during the basal event, the animals were in lacto-acidemia, since it was observed that there was a statistically significant decrease in lactate levels by 7%, 11%, and 17% at 1 h, 6 h, and 24 h, respectively, in G_2_ and G_3_ (*p* = 0.001). In G_4_, decreases of 13% at 1 h, 20% at 6 h, and 55% at 24 h were observed (*p* = 0.001).

### 3.3. Thermal Response

Table 3 shows the temperature differences of the four thermal windows (OCU, EAR, NOSE, LIMB). Regarding OCU, in all groups where caffeine was administered, the surface temperature was 0.15 °C higher compared to G_1_ at 6 h (*p* = 0.04) and 0.98 °C higher compared to G_1_ at 24 h (*p* = 0.0001). Likewise, it was found that G_2_ and G_1_ presented a general increase of 1.20 °C at 6 h, and of 1.45 °C at 24 h (*p* = 0.001). In G_4_, this increase was 1.25 °C at 6 h and 2.22 °C at 24 h (*p* = 0.001).

For EAR, it was observed that the G_4_ temperature was higher by 0.76 °C and 1 °C compared to G_3_ and G_1_, respectively *(p* = 0.0005). Between evaluation times, all treatments presented statistically significant decreases of up to 2 °C at 1 h, and a statistically significant increase of up to 2.7 °C at 6 h. At 24 h, G_4_ had the greatest statistically significant increase of 2.41 °C compared to basal (*p* = 0.001).

At NOSE, it was observed that G_4_ was statistically 2.62 °C higher compared to G_3_, 3.09 °C compared to G_2,_ and 5.47 °C compared to G_1_ at 24 h (*p* = 0.0001). Between evaluation times, all groups presented a statistically significant decrease of 2.7 °C at 1 h. Subsequently, there was a statistically significant increase of 2.8 °C at 6 h, while at 24 h, a significant increase of 6.3 °C and 7.12 °C was observed in G_2_ and G_3_, respectively. Nonetheless, G_4_ had the highest temperatures (32.29 ± 0.05 °C) and a statistically significant increase of 9.74 °C (*p* = 0.001).

Regarding LIMB, Table 3 shows that G_4_ piglets had the highest temperatures at 24 h (33.49 ± 0.11 °C), differing by 2.11 °C from G_2_ and by 1.79 °C from G_3_ (*p* = 0.001). Between evaluation times, all treatments showed a temperature decrease from basal to 1 h of up to 2.62 °C. In contrast, from 1 h to 24 h, a progressive increase of 3.48–8.7 °C was registered in G_1_, G_2_, G_3_, and G_4_ (*p* = 0.001), where G_4_ showed the greatest increase, from 24.79 ± 0.11 °C to 33.49 ± 0.11 °C (*p* = 0.001).

RT values are shown in Table 4, where at 24 h, G_4_ was 0.58 °C higher in comparison to G_3_, 0.66 °C higher than G_2,_ and 0.7 °C higher than G_1_ (*p* = 0.004). Regarding the differences between evaluation times, G_4_ presented the greatest increase in temperature at 1 h, 6 h, and 24 h, registering statistically significant increases of 0.13 °C, 0.14 °C, and 0.8 °C, respectively (*p* = 0.001).

Table 5 summarizes the correlations between the different thermal windows and RT. A weak positive correlation was observed for OCU (*r* = 0.26, *p* = 0.0001), EAR (*r* = 0.29, *p* = 0.0001), and LIMB (*r* = 0.33, *p* = 0.0001), while NOSE had an intermediate positive correlation (*r* = 0.41, *p* = 0.0001). 

## 4. Discussion

The results show that the administration of caffeine at a dose of 30 mg/kg (G_4_) caused the most significant effects in low-birthweight and low-vitality newborn piglets, suggesting this dose as the more recommended one to improve the physiological, metabolic, thermal, and acid–base balance profile, in contrast to placebo (G_1_), low dose (10 mg/kg) (G_2_), and medium dose (20 mg/kg) (G_3_). However, the benefits observed in all groups receiving caffeine show that, regardless of the dose, caffeine is a neuroprotective drug against the consequences of neonatal hypoxia-ischemia events.

The possible explanation for the differences in the therapeutic effect between the three doses is related to the pharmacokinetic characteristics of caffeine. Menozzi et al. [29] evaluated the pharmacokinetics of caffeine in sows receiving 25 mg/kg, finding that the maximum concentration (C_Max_) was 20.02 ± 1.51 mg/mL at 9.51 h post-administration. Superchi et al. [30] reported that PO administration of caffeine at 27 mg/kg to parturient sows did not affect physiological parameters or behavior, although it reduced stillborn percentages. A similar result has been reported in sheep receiving 10 or 20 mg/kg of caffeine in Robertson et al. [31]’s study, where no significant effect on newborn survival or physiological parameters, such as RT, was reported. The authors attribute this variation to the differences in caffeine metabolism and distribution when comparing therapeutic and toxic ranges between humans and neonatal animals [38].

These results suggest that a lower dose of caffeine (10 or 20 mg/kg) would take longer to reach peak plasma concentrations and, consequently, have a possible delayed therapeutic effect in animals. Moreover, studies in neonate humans have shown that plasma half-life and metabolism of caffeine are longer compared with adults or animals [39]. In this sense, Murdock et al. [28] analyzed the optimal concentration and duration of caffeine to improve lamb viability, finding that 20 mg/kg administered at day 120 of gestation increased lambs’ RT, as well as the attempts to suck colostrum and suction time. Accordingly, caffeine metabolism in the newborn can be considered less efficient due to a lower expression of the CYP1A2 protein enzymes, responsible for drug biotransformation, resulting in lower production of active metabolites such as theophylline and theobromine, the main caffeine-derived effectors. Therefore, although caffeine at 10 or 20 mg/kg might have a therapeutic effect in the newborn piglet (G_2_ and G_3_), the effect is limited and lasts for a short time, as observed in the results of this study.

In the present research, the use of caffeine at a dose of 30 mg/kg in G_4_ piglets significantly improved the alterations observed in the physio-metabolic profile associated with hypoxia and hypothermia. Regardless of the treatment group, all animals presented blood gas imbalances such as respiratory acidosis, reduced pO_2_, and hypercapnia at basal time before caffeine administration. Robertson et al. [40] mention that the main cause of acidemia in animals is hypercapnia, which is a recurrent event in newborn piglets. Swinbourne et al. [2] describe that the decrease in maternal blood flow to the fetus significantly affects gas exchange, leading to suboptimal O_2_ levels, elevated pCO_2_ concentrations, and respiratory acidosis. The decrease in blood pH triggers compensatory mechanisms such as the formation of HCO_3_^−^ when CO_2_ and H_2_O are metabolized by carbonic anhydrase [41,42]. It is known that caffeine stimulates the respiratory centers, improving gas exchange, increasing the tidal volume and HCO_3_^−^ excretion at the renal level, helping to correct the acid–base imbalances in the newborn [32,43,44]. Thus, as observed in piglets from G_4_, caffeine could have a protective effect against ischemia caused by hypoxia and acidemia [22,45,46].

Additionally, the administration of caffeine reduced pCO_2_ and increased pO_2_ levels, which has beneficial effects by improving vitality scores and survival rates in piglets. The antagonism of caffeine on A2a and A2b adenosine receptors present in pulmonary and cerebral tissue [47,48] stimulates the medullary respiratory centers, increasing sensitivity to CO_2_ concentrations and improving respiratory function [26,49]. In the same way, stimulation of the central *vagus* nerve and the *medulla oblongata* help increase tidal volume, which would explain the increase in O_2_ levels and the reduction in CO_2_ [24,50]. According to this respiratory and physiological response, it can be suggested that high doses of caffeine (30 mg/kg) could be the best to improve the ventilatory activity of newborn piglets. The higher improvement observed with this dose is related to the pharmacokinetic properties of caffeine and that at high doses, plasmatic concentrations of the drug can be found even at 9 h after its administration [31]. 

Regarding the respiratory effect of caffeine and its impact on pCO_2_ and pO_2_ levels, the prepartum administration of caffeine in sows and its effect on piglets [51] and newborn lambs [52] has shown that caffeine lowers the incidence of hypoxia due to a short-term physio-metabolic and behavioral adaptation during birth, avoiding cerebral ischemic damage and improving the performance of the piglets during feeding and lactation. It is worth mentioning that since the pH levels depend on pCO_2_ and pO_2_ levels, lactate is a biomarker that is also associated with neonatal acidosis. Van Dijk et al. [53] explains that the decrease in oxygen supply, apart from reducing blood pH, also leads to states of metabolic acidosis due to the increase in the production of lactic acid, as could be observed in the basal events of all the treatments. In piglets suffering from fetal hypoxia, the production of lactic acid is an alternative anaerobic energy pathway that leads to an inefficient production of ATP [21,54]. As found in G_2_, G_3_, and G_4_, caffeine reduced lactate concentrations at 1 h, 6 h, and 24 h, suggesting that it can restore aerobic pathways for energy production.

The observed results also showed that caffeine significantly increased glucose, obtaining the most significant increase at 24 h in G_4_ (*p* = 0.0001). Perhaps, this is one of the most important effects of caffeine related to vitality. The increase in glucose observed in all groups receiving caffeine is due to the sympathetic stimulation, which allows the stimulation of the chromaffin cells of the adrenal medulla that consequently release adrenaline, leading to an increase in blood glucose due to hepatic glycogenolysis and gluconeogenesis [55]. The effect that caffeine has on glucose levels has been controversial among authors because it has been seen in previous reports that caffeine in newborn piglets has not shown a positive increasing effect in glucose. However, the same authors describe that a limitation could be the administration of caffeine prior to colostrum intake [10,27]. In humans, it has been seen that fasting before caffeine administration reduces the body’s sensitivity to insulin, thus increasing blood glucose. The above may be due to the effect of adrenaline and glucagon, which promote the release of glucose stored in the liver, thus increasing blood glucose levels [56]. 

It would be worth adding that, according to the findings of other authors, caffeine treatment cannot replace colostrum intake, as colostrum is a valuable energy resource that is essential to thermoregulate, increase vitality, and obtain the therapeutic effect of caffeine [57]. Moreover, apart from colostrum and caffeine dose, birthweight highly influences the effect that this drug has on newborn piglets [27]. Thus, when considering caffeine as a neonatal drug for piglets, both the dose and the weight of the animal need to be considered to start a therapeutic protocol, as shown in the present study where a dose of 30 mg/kg could be considered an appropriate dose for piglets below 1 kg [10]. 

Regarding Ca^++^ values, caffeine administration at three doses significantly reduced Ca^++^ concentration at 24 h, especially in G_4_. It has been reported that hypoxia alters calcium metabolism in animals [58] and that caffeine is known to reduce Ca++ concentrations by reducing renal and intestinal reabsorption [59]. However, in the present study, the decrease in Ca^++^ might suggest an adaptative mechanism to hypoxia in response to caffeine and its stimulant cardiovascular effects. Caffeine facilitates intracellular Ca^++^ mobilization to improve muscular performance and cardiac activity [60]. In vitro studies have shown that caffeine has benefits on cardiac contractility [61], possibly improving oxygen transportation to other cellular tissues in hypoxic piglets. This was reported in Merino newborn lambs, in which 20 mg/kg of caffeine administration reduced neonatal mortality in the first week post-farrowing due to hypoxia reduction. Moreover, the increased availability of intracellular Ca^++^ might also be used by piglets to increase their body temperature through muscle shivering or muscle-based non-shivering thermogenesis through sarcoendoplasmic reticulum calcium [62]. 

Regarding the thermal response in different thermal windows and on the RT of piglets, the progressive increases in OCU, EAR, NOSE, LIMB, and RT are due to caffeine boosting basal metabolism by interacting with adenosine receptors in the sympathetic nervous system [63]. Jarrat et al. [10] mentions that raising metabolic activity in newborn piglets that have limited energy resources at birth can help them increase body temperature [13]. When comparing the administration of caffeine alone or in combination with glucose, the combination prolonged energy intake and improved the thermoregulatory capacity of newborn piglets [10]. 

OCU reflects the sympathetic influence on the surface temperature because it is a region where blood supply from the infra-orbital artery is mediated by sympathetic fibers from the facial nerve. Therefore, the change in the thermal response could be an indirect way to assess sympathetic activation [20,57], as shown in bovines [64] and canids [65]. On the other hand, NOSE temperature is related to the respiratory activity of piglets, keeping a positive correlation with respiratory rate and heat loss—or the amount of heat radiated through this thermal window [66,67]. Considering that caffeine stimulates cardiorespiratory function, the administration of caffeine in G4 induced the greatest increase in NOSE values, possibly reflecting a positive correlation between respiratory rate and thermal exchange.

Finally, the improved metabolic activity in G_2_, G_3_, and G_4_—see glucose discussion—is related to RT and the different thermal windows [63]. In addition, although it was not the main objective of the present study, it was possible to observe a difference between the thermal response of peripheral regions (LIMB) and central thermal windows (OCU, EAR, and NOSE). This could indicate that in hypothermic newborn piglets, blood microcirculation is shifted to vital body regions, as observed in other species [64,65]. Additionally, although the present study did not directly assess the effect of caffeine doses on the mortality rate of piglets (which could be considered a limitation of the study), further research needs to consider the effect that caffeine has on mortality rate and its association with blood gas parameters, physio-metabolic profile, and thermal response of piglets. To this extent, the findings of the present research suggest that IRT, along with blood biomarkers, could be a tool for monitoring the health status of newborn piglets [20]. Nonetheless, future research is needed.

## 5. Conclusions

According to the results, the administration of caffeine at 30 mg/kg reduced the alterations observed in piglets with low birthweight and low vitality scores. This was observed as a decrease in pCO_2_ levels and increasing pO_2_, which allows the recovery of the acid–base balance by increasing blood pH. Likewise, the administration of this drug enables a reduction in metabolic imbalances thanks to a reduction in lactic acid values and an increase in blood glucose. Finally, this drug increases infrared superficial thermal response capacity in the nasal, ocular, auricular, and nasal thermal windows in newborn piglets with low birthweight and low vitality scores. Further studies could consider the parameters evaluated in the present study and compare animals with low birthweight and low vitality scores days after farrowing with healthy piglets. Therefore, according to the attenuation of the metabolic, acid–base, and thermal changes observed in newborn piglets, it could be suggested that the use of high doses of caffeine (30 mg/kg) can improve the physiological response of piglets.

These results suggest that the administration of caffeine is a useful strategy to recover low vitality birth piglets, which is important for improving animal welfare and performance in the pig production system.

## Figures and Tables

**Table 1:** Value of blood gas parameters (mean ± SE) of newborn piglets with low birthweight and low vitality scores receiving different oral doses of caffeine.

Parameter	Treatment	Basal	1 h	6 h	24 h	*p*-Value
pH	G_1_ (Saline)	7.20 ± 0.005 ^a,3^	7.23 ± 0.005 ^c,2^	7.22 ± 0.005 ^b,2^	7.25 ± 0.005 ^b,c,1^	0.006
	G_2_ (10 mg/kg)	7.19 ± 0.008 ^a,3^	7.26 ± 0.005 ^b,2^	7.29 ± 0.005 ^a,1^	7.24 ± 0.008 ^b,c,2^	0.001
	G_3_ (20 mg/kg)	7.21 ± 0.005 ^a,3^	7.28 ± 0.005 ^a,2^	7.30 ± 0.005 ^a,1^	7.27 ± 0.005 ^b,2^	0.01
	G_4_ (30 mg/kg)	7.17 ± 0.006 ^a,3^	7.29 ± 0.005 ^a,2^	7.30 ± 0.005 ^a,2^	7.33 ± 0.006 ^a,1^	0.001
	*p*-value	0.91	0.0001	0.001	0.0001	
pCO_2_ (mmHg)	G_1_ (Saline)	85.93 ± 0.63 ^a,1^	82.93 ± 0.63 ^a,2^	80.93 ± 0.63 ^a,3^	79.93 ± 0.63 ^a,4^	0.001
	G_2_ (10 mg/kg)	85.08 ± 0.75 ^a,1^	81.93 ± 0.63 ^b,2^	79.93 ± 0.63 ^b,4^	83.00 ± 0.74 ^a,3^	0.005
	G_3_ (20 mg/kg)	85.25 ± 0.48 ^a,1^	80.93 ± 0.63 ^b,2^	77.93 ± 0.63 ^c,2^	77.13 ± 0.47 ^a,2^	0.002
	G_4_ (30 mg/kg)	87.46 ± 0.58 ^a,1^	77.93 ± 0.63 ^c,2^	76.95 ± 0.63 ^c,2^	60.43 ± 0.59 ^b,3^	0.001
	*p*-value	0.87	0.0001	0.001	0.001	
pO_2_ (mmHg)	G_1_ (Saline)	16.83 ± 0.36 ^a,2^	17.83 ± 0.36 ^c,1^	17.84 ± 0.36 ^a,1^	17.83 ± 0.75 ^b,1^	0.0001
	G_2_ (10 mg/kg)	16.98 ± 0.31 ^a,2^	19.83 ± 0.36 ^a,1^	17.83 ± 0.36 ^a,2^	18.00 ± 0.31 ^b,1^	0.0001
	G_3_ (20 mg/kg)	15.82 ± 0.21 ^a,2^	18.83 ± 0.36 ^b,1^	18.84 ± 0.36 ^b,1^	19.82 ± 0.21 ^a,1^	0.0001
	G_4_ (30 mg/kg)	16.44 ± 0.17 ^a,3^	18.82 ± 0.36 ^b,2^	18.83 ± 0.36 ^b,2^	21.06 ± 0.23 ^a,1^	0.0001
	*p*-value	0.52	0.0001	0.0001	0.0005	
HCO_3−_ (mmol/L)	G_1_ (Saline)	18.35 ± 0.22 ^a,1^	18.36 ± 0.22 ^b,1^	18.34 ± 0.22 ^b,1^	18.37 ± 0.22 ^d,1^	0.99
	G_2_ (10 mg/kg)	18.20 ± 0.16 ^a,2^	18.36 ± 0.22 ^b,2^	18.35 ± 0.22 ^b,2^	19.42 ± 0.16 ^c,1^	0.001
	G_3_ (20 mg/kg)	18.26 ± 0.18 ^a,2^	18.36 ± 0.22 ^b,2^	18.44 ± 0.22 ^b,2^	20.47 ± 0.18 ^b,1^	0.001
	G_4_ (30 mg/kg)	18.22 ± 0.15 ^a,3^	18.50 ± 0.22 ^a,3^	19.50 ± 0.22 ^a,2^	22.22 ± 0.15 ^a,1^	0.001
	*p*-value	0.89	0.002	0.0001	0.0001	

^a,b,c,d^ Different letters indicate significant differences between treatments in the same evaluation time. ^1,2,3,4^ Different numbers indicate significant differences between evaluation times in a single treatment. *n* = 120 piglets per each group. G_1_: placebo (saline); G_2_: low dose (10 mg/kg); G_3_: medium dose (20 mg/kg); G_4_: high dose (30 mg/kg).

**Table 2:** Values of physio-metabolic parameters (mean ± SE) of newborn piglets with low birthweight and low vitality receiving different oral doses of caffeine.

Parameter	Treatment	Basal	1 h	6 h	24 h	*p*-Value
Ca (mmol/L)	G_1_ (Saline)	2.21 ± 0.02 ^a,1^	2.10 ± 0.02 ^b,2^	2.09 ± 0.02 ^b,2^	2.08 ± 0.02 ^d,2^	0.0001
	G_2_ (10 mg/kg)	2.16 ± 0.01 ^a,1^	2.09 ± 0.02 ^b,1^	2.08 ± 0.02 ^b,1^	2.02 ± 0.01 ^c,2^	0.001
	G_3_ (20 mg/kg)	2.17 ± 0.01 ^a,1^	2.07 ± 0.02 ^a,2^	2.06 ± 0.02 ^a,2^	1.92 ± 0.01 ^b,3^	0.03
	G_4_ (30 mg/kg)	2.08 ± 0.01 ^a,1^	2.05 ± 0.02 ^a,1^	2.05 ± 0.02 ^a,1^	1.80 ± 0.01 ^a,2^	0.0001
	*p*-value	0.92	0.0001	0.006	0.002	
Glucose (mg/dL)	G_1_ (Saline)	58.36 ± 0.70 ^a,3^	65.36 ± 0.70 ^d,2^	66.36 ± 0.70 ^d,1^	67.36 ± 0.70 ^c,1^	0.0001
	G_2_ (10 mg/kg)	55.88 ± 0.68 ^a,3^	67.36 ± 0.70 ^c,1^	67.36 ± 0.70 ^c,1^	64.88 ± 0.70 ^d,2^	0.001
	G_3_ (20 mg/kg)	56.65 ± 1.25 ^a,3^	68.36 ± 0.70 ^b,2^	70.36 ± 0.70 ^b,1^	69.65 ± 0.70 ^b,1^	0.0001
	G_4_ (30 mg/kg)	57.08 ± 1.18 ^b,4^	69.36 ± 0.75 ^a,3^	72.36 ± 0.70 ^a,2^	97.33 ± 0.70 ^a,1^	0.02
	*p*-value	0.95	0.0001	0.0001	0.007	
Lactate (mg/dL)	G_1_ (Saline)	93.97 ± 0.76 ^a,1^	91.97 ± 0.76 ^a,2^	91.97 ± 0.76 ^a,2^	89.97 ± 0.76 ^a,3^	0.0001
	G_2_ (10 mg/kg)	96.48 ± 2.09 ^a,1^	90.97 ± 0.76 ^b,1^	89.97 ± 0.76 ^b,1^	84.44 ± 2.09 ^b,2^	0.001
	G_3_ (20 mg/kg)	92.67 ± 0.79 ^a,1^	84.97 ± 0.76 ^d,2^	82.97 ± 0.76 ^c,3^	75.28 ± 0.79 ^c,4^	0.001
	G_4_ (30 mg/kg)	94.69 ± 0.69 ^a,1^	82.97 ± 0.76 ^c,2^	74.97 ± 0.76 ^d,3^	41.69 ± 0.69 ^d,4^	0.001
	*p*-value	0.99	0.001	0.0001	0.006	

^a,b,c,d^ Different letters indicate significant differences between treatments in the same evaluation time. ^1,2,3^ Different numbers indicate significant differences between evaluation times in a single treatment. *n* = 120 piglets per each group. G_1_: placebo (saline); G_2_: low dose (10 mg/kg); G_3_: medium dose (20 mg/kg); G_4_: high dose (30 mg/kg).

**Table 3:** Temperature values of the four thermal windows (OCU, EAR, NOSE, LIMB) (°C) (mean ± SE) of newborn piglets with low birthweight and low vitality receiving different oral doses of caffeine.

Thermal Window	Treatment	Basal	1 h	6 h	24 h	*p*-Value
OCU	G_1_ (Saline)	33.63 ± 0.03 ^a,2^	32.04 ± 0.03 ^c,3^	34.65 ± 0.03 ^b,1^	34.79 ± 0.03 ^b,1^	0.001
	G_2_ (10 mg/kg)	33.84 ± 0.03 ^a,3^	32.14 ± 0.03 ^b,4^	34.78 ± 0.03 ^a,2^	35.00 ± 0.03 ^a,1^	0.001
	G_3_ (20 mg/kg)	33.58 ± 0.02 ^a,3^	32.27 ± 0.02 ^a,4^	34.79 ± 0.02 ^a,2^	35.03 ± 0.02 ^a,1^	0.001
	G_4_ (30 mg/kg)	33.55 ± 0.02 ^a,3^	32.30 ± 0.02 ^a,4^	34.80 ± 0.02 ^a,2^	35.77 ± 0.02 ^a,1^	0.001
	*p*-value	0.96	0.001	0.04	0.0001	
EAR	G_1_ (Saline)	32.54 ± 0.03 ^a,3^	30.07 ± 0.03 ^a,4^	33.66 ± 0.03 ^a,2^	33.91 ± 0.03 ^c,1^	0.0001
	G_2_ (10 mg/kg)	32.58 ± 0.03 ^a,3^	30.24 ± 0.03 ^a,4^	33.73 ± 0.03 ^a,2^	33.96 ± 0.03 ^c,1^	0.001
	G_3_ (20 mg/kg)	32.60 ± 0.03 ^a,3^	30.30 ± 0.02 ^b,4^	33.79 ± 0.02 ^a,2^	34.14 ± 0.02 ^b,1^	0.001
	G_4_ (30 mg/kg)	32.49 ± 0.03 ^a,3^	30.36 ± 0.03 ^b,4^	33.76 ± 0.03 ^a,2^	34.90 ± 0.03 ^a,1^	0.001
	*p*-value	0.97	0.005	0.37	0.0005	
NOSE	G_1_ (Saline)	22.62 ± 0.11 ^a,3^	19.91 ± 0.11 ^a,4^	23.30 ± 0.11 ^b,2^	26.82 ± 0.11 ^d,1^	0.001
	G_2_ (10 mg/kg)	22.70 ± 0.10 ^a,3^	19.97 ± 0.10 ^a,4^	23.62 ± 0.10 ^a,b,2^	29.02 ± 0.10 ^c,1^	0.001
	G_3_ (20 mg/kg)	22.55 ± 0.07 ^a,3^	19.94 ± 0.07 ^a,4^	23.76 ± 0.07 ^a,2^	29.67 ± 0.07 ^b,1^	0.001
	G_4_ (30 mg/kg)	22.53 ± 0.05 ^a,3^	20.08 ± 0.05 ^a,4^	23.96 ± 0.05 ^a,2^	32.29 ± 0.05 ^a,1^	0.001
	*p*-value	0.88	0.69	0.0001	0.0001	
LIMB	G_1_ (Saline)	27.19 ± 0.11 ^a,3^	24.57 ± 0.11 ^a,4^	28.05 ± 0.11 ^a,2^	30.42 ± 0.11 ^c,1^	0.001
	G_2_ (10 mg/kg)	27.25 ± 0.11 ^a,3^	24.64 ± 0.11 ^a,4^	28.30 ± 0.11 ^a,2^	31.38 ± 0.11 ^b,1^	0.001
	G_3_ (20 mg/kg)	27.06 ± 0.11 ^a,3^	24.83 ± 0.11 ^a,4^	28.41 ± 0.11 ^a,2^	31.70 ± 0.11 ^b,1^	0.001
	G_4_ (30 mg/kg)	27.14 ± 0.11 ^a,3^	24.79 ± 0.11 ^a,4^	28.62 ± 0.11 ^a,2^	33.49 ± 0.11 ^a,1^	0.001
	*p*-value	0.93	0.95	0.60	0.001	

^a,b,c,d^ Different letters indicate significant differences between treatments in the same evaluation time. ^1,2,3,4^ Different numbers indicate significant differences between evaluation times in a single treatment. *n* = 120 piglets per each group. G_1_: placebo (saline); G_2_: low dose (10 mg/kg); G_3_: medium dose (20 mg/kg); G_4_: high dose (30 mg/kg).

**Table 4:** RT (°C) (mean ± SE) of newborn piglets with low birthweight and low vitality receiving different oral doses of caffeine.

Treatment	Basal	1 h	6 h	24 h	*p*-Value
G_1_ (Saline)	36.18 ± 0.02 ^a,2^	36.18 ± 0.01 ^a,2^	36.27 ± 0.02 ^a,1^	36.28 ± 0.03 ^c,1^	0.001
G_2_ (10 mg/kg)	36.18 ± 0.01 ^a,2^	36.18 ± 0.02 ^a,2^	36.27 ± 0.01 ^a,1^	36.28 ± 0.02 ^b,1^	0.001
G_3_ (20 mg/kg)	36.12 ± 0.02 ^a,1^	36.18 ± 0.01 ^a,2^	36.27 ± 0.02 ^a,2^	36.42 ± 0.01 ^b,3^	0.001
G_4_ (30 mg/kg)	36.14 ± 0.03 ^a,1^	36.27 ± 0.02 ^b,2^	36.28 ± 0.01 ^a,2^	36.94 ± 0.01 ^a,3^	0.001
*p*-value	0.85	0.001	0.98	0.004	

^a,b,c^ Different letters indicate significant differences between treatments in the same evaluation time. ^1,2,3^ Different numbers indicate significant differences between evaluation times in a single treatment. *n* = 120 piglets per each group. G_1_: placebo (saline); G_2_: low dose (10 mg/kg); G_3_: medium dose (20 mg/kg); G_4_: high dose (30 mg/kg).

**Table 5:** Correlation matrix.

	OCU	NOSE	EAR	LIMB	RT
OCU	1.00	0.68 *p* = 0.0001	0.92 *p* = 0.0001	0.76 *p* = 0.0001	0.26 *p* = 0.0001
NOSE	0.68 *p* = 0.0001	1.00	0.79 *p* = 0.0001	0.84 *p* = 0.0001	0.41 *p* = 0.0001
EAR	0.92 *p* = 0.0001	0.79 *p* = 0.0001	1.00	0.79 *p* = 0.0001	0.29 *p* = 0.0001
LIMB	0.76 *p* = 0.0001	0.84 *p* = 0.0001	0.79 *p* = 0.0001	1.00	0.33 *p* = 0.0001
RT	0.26 *p* = 0.0001	0.41 *p* = 0.0001	0.29 *p* = 0.0001	0.33 *p* = 0.0001	1.00

OCU = periocular area; EAR = auricular region; NOSE = nasal; LIMB = left hindlimb region; RT = rectal temperature.

## Data Availability

The data presented in this study are available on request from the corresponding author. The data are not publicly available due to the original data belonging to the swine breeding herd.
